# *Salicornia* as a crop plant in temperate regions: selection of genetically characterized ecotypes and optimization of their cultivation conditions

**DOI:** 10.1093/aobpla/plu071

**Published:** 2014-11-10

**Authors:** Devesh Singh, Anne K. Buhmann, Tim J. Flowers, Charlotte E. Seal, Jutta Papenbrock

**Affiliations:** 1 Institute of Botany, Leibniz University Hannover, D-30419 Hannover, Germany; 2 Department of Environmental Sciences—Botany, Basel University, Schönbeinstrasse 6, CH-4056 Basel, Switzerland; 3 School of Life Sciences, University of Sussex, Johnik Maynard Smith Building, Falmer, Brightons BN1 9QG, UK; 4 Seed Conservation Department, Royal Botanic Gardens Kew, Wakehurst Place, Ardingly, West Sussex RH17 6TN, UK

**Keywords:** Biomass, ETS, germination, *Salicornia* species, salinity.

## Abstract

Rising sea levels and salinization of groundwater due to global climate change result in fast-dwindling sources of freshwater. Therefore, it is important to find alternatives to grow food crops and vegetables. Halophytes are naturally evolved salt-tolerant plants that are adapted to grow in environments that inhibit the growth of most glycophytic crop plants substantially. Members of the Salicornioideae are promising candidates for saline agriculture due to their high tolerance to salinity. Our aim was to develop genetically characterized lines of *Salicornia* and *Sarcocornia* for further breeding and to determine optimal cultivation conditions. To obtain a large and diverse genetic pool, seeds were collected from different countries and ecological conditions. The external transcribed spacer (ETS) sequence of 62 *Salicornia* and *Sarcocornia* accessions was analysed: ETS sequence data showed a clear distinction between the two genera and between different *Salicornia* taxa. However, in some cases the ETS was not sufficiently variable to resolve morphologically distinct species. For the determination of optimal cultivation conditions, experiments on germination, seedling establishment and growth to a harvestable size were performed using different accessions of *Salicornia* spp. Experiments revealed that the percentage germination was greatest at lower salinities and with temperatures of 20/10 °C (day/night). *Salicornia* spp. produced more harvestable biomass in hydroponic culture than in sand culture, but the nutrient concentration requires optimization as hydroponically grown plants showed symptoms of stress. *Salicornia ramosissima* produced more harvestable biomass than *Salicornia dolichostachya* in artificial sea water containing 257 mM NaCl. Based on preliminary tests on ease of cultivation, gain in biomass, morphology and taste, *S. dolichostachya* was investigated in more detail, and the optimal salinity for seedling establishment was found to be 100 mM. Harvesting of *S. dolichostachya* twice in a growing season was successful, but the interval between the harvests needs to be optimized to maximize biomass production.

## Abstract (precis)

Salinization of groundwater results in fast dwindling sources of freshwater. Our aim was to develop genetically characterized lines of the salt-tolerant *Salicornia* (marsh samphire) and *Sarcocornia* (shrubby Swampfire) as new crop plants. To obtain a large genetic pool, seeds were collected from different countries and ecological conditions. The application of a genetic marker showed a clear distinction between the two genera and between 57 *Salicornia* taxa. For the determination of optimal cultivation conditions, experiments on germination, seedling establishment and growth to a harvestable size were performed using different *Salicornia* accessions. Further optimization of cultivation conditions is necessary for commercial use.

## Introduction

The area of arable land under cultivation is decreasing due to global climatic changes that are resulting from rising sea levels and salinization of groundwater. At the same time, the human population of the world is increasing so that terrestrial biomass production is of great importance for the delivery of food and feedstocks (FAO 2011). The development of specialized saline agriculture offering high-value products is a serious alternative to conventional agriculture relying on non-saline soils and freshwater. In saline agriculture, saline water is considered to be a resource rather than a threat, thus opening a further opportunity for producing biomass and other bio-products. Our goal is to foster the domestication and breeding of salt-tolerant plants, improving their agricultural traits and developing their potential as food varieties. In order to achieve this goal, genetically defined lines of halophytic species and standardized cultivation conditions need to be established.

Halophytes are believed to have arisen many times in unrelated plant families during the evolution of angiosperms (Bennett *et al.* 2013) although currently they comprise, at most, 2 % of terrestrial plant species. However, they include a wide diversity of plant forms and have been utilized as vegetable, forage and oilseed crops. The genus *Salicornia* in the Amaranthaceae has generated significant interest as a multi-purpose plant. It is suitable for cultivation as a vegetable in highly saline environments (Ventura *et al.* 2010, 2011*a*) and as a source of valuable secondary compounds (Buhmann and Papenbrock 2013*b*).

*Salicornia* species are succulent annuals with extremely reduced leaves and a spike-like terminal inflorescence (Scott 1977). The genus, which is widely dispersed in Eurasia, North America and South Africa, presently comprises around 25–30 species (Kadereit *et al.* 2007). The poorly characterized taxonomy, which makes it extremely difficult to classify most species, microspecies, subspecies, varieties and putative hybrids (Kadereit *et al.* 2007; Teege *et al.* 2011), is a major constraint to the use of *Salicornia* spp. as a crop. The paucity of morphological characters, phenotypic plasticity, breeding systems and hybridizations are all contributory factors (Ball and Akeroyd 1993). Consequently, it is very difficult to recognize whether plants that occur in the same area and share a similar morphology have the same genotype. Molecular sequence data show that the taxonomic uncertainty in *Salicornia* spp. has two major causes: (i) in the presence of high phenotypic plasticity, the same genotypes have been named differently in different regions, and (ii) striking morphological parallelism and weak morphological differentiation have led to the misapplication of the same name to different genotypes (Kadereit *et al.* 2007).

Three basic conditions must be satisfied if *Salicornia* spp*.* are to be used successfully as an irrigated crop: (i) high yield potential; (ii) extractable products must be a substitute for conventional plant products; and (iii) the irrigation requirements should be in the range of those used for conventional crops and cause no damage to the soil. *Salicornia* spp. can be grown in insulated wetland systems and irrigated with nutrient-rich saline sewage water or effluents from aquaculture (i.e. as a biofilter) to increase sustainability (Brown *et al.* 1999; Buhmann and Papenbrock 2013*a*). For commercial production, all steps of cultivation need to be controllable, predictable and projectable. Therefore, the aim of this study was to characterize *Salicornia* species genetically. We used ETS sequence data, an established marker for the characterization of the *Salicornia* group, to define and recognize species, subspecies and genotypes (Kadereit *et al.* 2007). Additionally, optimal conditions for germination, seedling establishment and plant cultivation were determined for several genotypes. The objective was to increase the performance of *Salicornia* spp*.* as an agronomic crop in highly saline environments.

## Methods

### Plant material

Seed material of *Salicornia* spp. and *Sarcocornia* spp. was obtained from five countries (Germany, the Netherlands, England, Israel and Kazakhstan; see Table 1). Seeds were collected from each of 62 individual plants along with material for a herbarium and for DNA extraction (both from 42 of the 62 plants): all samples were named with letters indicating the country of collection and numbers indicating the order of sampling (Table 1). In Germany, samples of *Salicornia* spp. or genotypes were collected along transects at four sites at Jade Bay in the following way: the distance (∼250 m) between plants of *Salicornia* spp. nearest to, and furthest from, the seashore (where they were not regularly flooded) was divided into five equally spaced points (50 m apart), from which samples were collected. The first sample (lowest number) was collected nearest to the seashore, and the last sample (highest number) furthest from the seashore (details of the sites, their locations and the species collected can be found in Table 1). In addition, seeds were collected for long-term storage from some of the populations to which the individuals belonged. All samples were analysed genetically and classified according to Kadereit *et al.* (2007).

**Table 1.** List of samples used in this study. The ETS sequences obtained were submitted to GenBank and the accession numbers are given. *S*., *Salicornia*; *Sa*., *Sarcocornia*.

No.	Place of collection	Coordinates	Species (ETS results)	Collected by	GenBank accession number
D1	Germany, Jade Bay, Caeciliengroden South	53°28′55″N; 8°03′18″E	*S. dolichostachya*	Buhmann, A.	KF427860
D2	Germany, Jade Bay, Caeciliengroden South	53°28′55″N; 8°03′18″E	*S. dolichostachya*	Buhmann, A.	KF427861
D3	Germany, Jade Bay, Caeciliengroden South	53°28′55″N; 8°03′18″E	*S. dolichostachya*	Buhmann, A.	KF427862
D4	Germany, Jade Bay, Caeciliengroden South	53°28′55″N; 8°03′18″E	*S. dolichostachya*	Buhmann, A.	KF427863
D5	Germany, Jade Bay, Caeciliengroden South	53°28′55″N; 8°03′18″E	*S. dolichostachya*	Buhmann, A.	KF427864
D6	Germany, Jade Bay, Caeciliengroden North	53°29′13″N; 8°03′16″E	*S. dolichostachya*	Buhmann, A.	KF427865
D7	Germany, Jade Bay, Caeciliengroden North	53°29′13″N; 8°03′16″E	*S. dolichostachya*	Buhmann, A.	KF427866
D8	Germany, Jade Bay, Caeciliengroden North	53°29′13″N; 8°03′16″E	*S. ramosissima*	Buhmann, A.	KF427867
D9	Germany, Jade Bay, Caeciliengroden North	53°29′13″N; 8°03′16″E	*S. ramosissima*	Buhmann, A.	KF427868
D10	Germany, Jade Bay, Idagroden	53°27′53″N; 8°03′50″E	*S. dolichostachya*	Buhmann, A.	KF427869
D11	Germany, Jade Bay, Idagroden	53°27′53″N; 8°03′50″E	*S. dolichostachya*	Buhmann, A.	KF427870
D12	Germany, Jade Bay, Idagroden	53°27′53″N; 8°03′50″E	*S. dolichostachya*	Buhmann, A.	KF427871
D13	Germany, Jade Bay, Idagroden	53°27′53″N; 8°03′50″E	*S. dolichostachya*	Buhmann, A.	KF427872
D14	Germany, Jade Bay, Idagroden	53°27′53″N; 8°03′50″E	*S. dolichostachya*	Buhmann, A.	KF427873
D15	Germany, Jade Bay, Idagroden	53°27′53″N; 8°03′50″E	*S. ramosissima*	Buhmann, A.	KF427874
D16	Germany, Jade Bay, Dangast	53°26′19″N; 8°09′49″E	*S. dolichostachya*	Buhmann, A.	KF427875
D17	Germany, Jade Bay, Dangast	53°26′19″N; 8°09′49″E	*S. dolichostachya*	Buhmann, A.	KF427876
D18	Germany, Jade Bay, Dangast	53°26′19″N; 8°09′49″E	*S. dolichostachya*	Buhmann, A.	KF427877
D19	Germany, Jade Bay, Dangast	53°26′19″N; 8°09′49″E	*S. dolichostachya*	Buhmann, A.	KF427878
D20	Germany, Jade Bay, Dangast	53°26′19″N; 8°09′49″E	*S. dolichostachya*	Buhmann, A.	KF427879
D21	Germany, Jade Bay, Dangast	53°26′19″N; 8°09′49″E	*S. ramosissima*	Buhmann, A.	KF427880
D22	Germany, Jade Bay, Dangast	53°26′19″N; 8°09′49″E	*S. ramosissima*	Buhmann, A.	KF427881
D23	Germany, Jade Bay, Dangast	53°26′19″N; 8°09′49″E	*S. ramosissima*	Buhmann, A.	KF427882
D24	Germany, Jade Bay, Dangast	53°26′19″N; 8°09′49″E	*S. ramosissima*	Buhmann, A.	KF427883
D25	Germany, Jade Bay, Dangast	53°26′19″N; 8°09′49″E	*S. dolichostachya*	Buhmann, A.	KF427884
D27	Germany, Jade Bay, Dangast	53°26′19″N; 8°09′49″E	*S. ramosissima*	Buhmann, A.	KF427885
D28	Germany, Jade Bay, Dangast	53°26′19″N; 8°09′49″E	*S. dolichostachya*	Buhmann, A.	KF427886
D29	Germany, Jade Bay, Dangast	53°26′19″N; 8°09′49″O	*S. dolichostachya*	Buhmann, A.	KF427887
NLD1	Netherlands, Wieringen	52°53′45″N; 4°54′43″E	*S. dolichostachya*	Buhmann, A., Katschnig, D.	KF427888
NLD2	Netherlands, Wieringen	52°53′45″N; 4°54′43″E	*S. dolichostachya*	Buhmann, A., Katschnig, D.	KF427889
NLD3	Netherlands, Wieringen	52°53′45″N; 4°54′43″E	*S. dolichostachya*	Buhmann, A. A., Katschnig, D.	KF427890
NLD4	Netherlands, Wieringen	52°53′45″N; 4°54′43″E	*S. dolichostachya*	Buhmann, A., Katschnig, D.	KF427891
NLD5	Netherlands, Wieringen	52°53′45″N; 4°54′43″E	*S. dolichostachya*	Buhmann, A., Katschnig, D.	KF427892
GB1	Great Britain, England, Guntner point	50°49′07.7″N 0°57′22.9″W	*S. dolichostachya*	Flowers, T., Streeter, D.	KF427893
GB2	Great Britain, England, Guntner point	50°49′07.7″N 0°57′22.9″W	*Sa. perennis*	Flowers, T., Streeter, D.	KF427894
GB3	Great Britain, England, Guntner point	50°49′07.7″N 0°57′22.9″W	*S. ramosissima*	Flowers, T., Streeter, D.	KF427895
GB4	Great Britain, England, Guntner point	50°49′07.7″N 0°57′22.9″W	*S. dolichostachya*	Flowers, T., Streeter, D.	KF427896
GB5	Great Britain, England, Guntner point	50°49′07.7″N 0°57′22.9″W	*S. dolichostachya*	Flowers, T., Streeter, D.	KF427897
GB6	Great Britain, England, Guntner point	50°49′07.7″N 0°57′22.9″W	*S. ramosissima*	Flowers, T., Streeter, D.	KF427898
GB7	Great Britain, England, Guntner point	50°49′07.7″N 0°57′22.9″W	*S. ramosissima*	Flowers, T., Streeter, D.	KF427899
GB8	Great Britain, England, Guntner point	50°49′07.7″N 0°57′22.9″W	*S. ramosissima*	Flowers, T., Streeter, D.	KF427900
GB9	Great Britain, England, Guntner point	50°49′07.7″N 0°57′22.9″W	*S. dolichostachya*	Flowers, T., Streeter, D.	KF427901
GB10	Great Britain, England, Guntner point	50°49′07.7″N 0°57′22.9″W	*S. dolichostachya*	Flowers, T., Streeter, D.	KF427902
GB11	Great Britain, England, Guntner point	50°49′07.7″N 0°57′22.9″W	*S. dolichostachya*	Flowers, T., Streeter, D.	KF427903
GB12	Great Britain, England, Guntner point	50°49′07.7″N 0°57′22.9″W	*S. ramosissima*	Flowers, T., Streeter, D.	KF427904
GB14	Great Britain, England, Guntner point	50°49′07.7″N 0°57′22.9″W	*S. ramosissima*	Papenbrock, J., Flowers, T.	KF427905
GB15	Great Britain, England, Newhaven	50°47′00.9″N 0°04′00.9″E	*S. ramosissima*	Papenbrock, J., Flowers, T.	KF427906
GB16	Great Britain, England, Newhaven	50°47′00.9″N 0°04′00.9″E	*S. ramosissima*	Papenbrock, J., Flowers, T.	KF427907
GB17	Great Britain, England, Newhaven	50°47′00.9″N 0°04′00.9″E	*S. ramosissima*	Papenbrock, J., Flowers, T.	KF427908
GB18	Great Britain, England, Newhaven	50°47′00.9″N 0°04′00.9″E	*S. ramosissima*	Papenbrock, J., Flowers, T.	KF427909
GB19	Great Britain, England, Newhaven	50°47′00.9″N 0°04′00.9″E	*S. ramosissima*	Papenbrock, J., Flowers, T.	KF427910
GB20	Great Britain, England, Newhaven	50°47′00.9″N 0°04′00.9″E	*S. ramosissima*	Papenbrock, J., Flowers, T.	KF427911
GB21	Great Britain, England, Newhaven	50°47′00.9″N 0°04′00.9″E	*S. dolichostachya*	Papenbrock, J., Flowers, T.	KF427912
GB22	Great Britain, England, Newhaven	50°47′00.9″N 0°04′00.9″E	*Sa. perennis*	Papenbrock, J., Flowers, T.	KF427913
GB23	Great Britain, England, Newhaven	50°47′00.9″N 0°04′00.9″E	*Sa. perennis*	Papenbrock, J., Flowers, T.	KF427914
RN	Israel, Dead sea area	Not known	*S. persica*	Ventura, Y., Sagi, M.	KF427915
N	Israel, Dead sea area	Not known	*S. persica*	Ventura, Y., Sagi, M.	KF427916
Kaz	Kazakhstan, High Saline area near the Aral sea	Not known	*S. perennans*	Ventura, Y., Sagi, M.	KF427917
DS	Israel, Dead sea area	Not known	*S. persica*	Ventura, Y., Sagi, M.	KF427918
IS	Israel, Ramat Negev Inland Salt Pan	Not known	*Sa. fruticosa*	Ventura, Y., Sagi, M.	KF427919
H4	Israel, exact location not known	Not known	*S. bigelovii*	Ventura, Y., Sagi, M.	KF427920
VM	Israel, Ramat Negev, Israel	Not known	*Sa. fruticosa*	Ventura, Y., Sagi, M.	KF427921

### Molecular marker analysis

Young stems of *Salicornia* spp. and *Sarcocornia* spp. of all samples listed in Table 1 were homogenized with a mortar and pestle in liquid nitrogen. DNA was extracted from the fine powdered plant material (100 mg) produced using the Plant Nucleospin II Kit (Macherey & Nagel, Düren, Germany) following the manufacturer's instructions. The region selected for PCR amplification was the nuclear ETS region. Primer pairs were P708 5′-CTCTAACTGATTTAATGAGCCATTCGCA-3′ and P707 5′-GTCCCTATTGTGTAGATTTCAT-3′ (Kadereit *et al.* 2007) to amplify a sequence of 672 bp. The total volume of 25 µL included 1× Dream *Taq* Green buffer, 0.2 mM dNTPs, 2 mM MgCl_2_, 1 U *Taq* polymerase (MBI Fermentas, St. Leon-Rot, Germany), 10–30 ng template DNA and 1 pmol of each primer. The PCR was performed in a PTC 200 thermocycler (Biozym-Diagnostik GmbH, Hess, Oldendorf, Germany) with a heated lid under the following conditions: initial denaturation 3 min at 95 °C followed by 30 cycles of denaturation of 30 s at 95 °C, primer annealing for 30 s at 47 °C and extension for 35 s at 72 C, terminated by a final hold for 8 min at 72 °C. All PCR were repeated two to four times independently from the same DNA to reduce the number of wrong base pairs in the final consensus sequence to a minimum due to errors produced by *Taq* polymerase. Direct sequencing of the PCR product was carried out by GATC Biotech (Konstanz, Germany).

Consensus sequences were obtained using Clone Manager 9 (Sci-Ed, Cary, NC, USA). These sequences were aligned by CLUSTAL X (Thompson *et al.* 1997) and the alignment was further modified by eye. Gaps were considered as missing data. For comparison, known ETS sequences of other *Salicornia* species from GenBank were added to the dataset [**see Supporting Information**]. Phylogenetic analysis was performed using the maximum likelihood (ML) algorithm with the general time reversal (GTR + G) model using MEGA version 5.1 (Tamura *et al.* 2011). In the analyses, trees were tested by the bootstrapping method with 1000 replications.

### Seed analysis and germination trials

Seeds from plants of six different collections (D11 and D16 to D20 from the transect and plants from two close locations) of *Salicornia* spp. were cleaned and separated by sieves of decreasing mesh size (to a minimum of 0.6 mm) before the debris was removed by aspiration. The seeds were then separated into small (<1.4 mm) and large seeds (≥1.5 mm) according to Ungar (1979) and mass per seed was determined.

Twenty small and large seeds from each of the six collections were used for a tetrazolium test to assess the initial seed viability. Seeds were imbibed in a Petri dish containing 1 % water-agar and incubated at 20 °C for 24 h. Some of the seeds germinated on the agar plate and were counted as viable seeds. After incubation, non-germinated seeds were sectioned carefully to expose the embryonic tissue. Sectioned seeds were immersed in 2,3,5-triphenyl tetrazolium chloride (1 %, w/v, aq.) and incubated in darkness at 30 °C for 48 h. After 48 h, seeds were rinsed with distilled water and observed under a stereoscopic microscope: highly stained seeds were considered as viable, while unstained, patchy or slightly stained seeds were scored as non-viable (ISTA 2012).

Seeds from the same collections were also used to conduct experiments investigating the influence of temperature and salinity on germination, but here seed samples consisted of a mixture of large and small seeds collected from the field. After drying to a moisture content of 15 %, the seeds were stored at 4 °C for 6 months before the germination experiments. To determine the influence of temperature on germination, seeds of three collections (D11, D16 and D20) were incubated at five temperatures with a 12-h photoperiod (constant temperatures of 5, 10 and 15 °C, and alternating temperatures of 15/5 and 20/10 °C where the higher temperature coincided with the light period). Three other collections (D17, D18 and D19) were incubated at 15/5 and 20/10 °C. To examine the influence of salinity on germination, seeds of all six collections of *Salicornia* spp. were subjected to six salinity regimes (0, 100, 200, 400, 700 and 1000 mM NaCl). Four replicates of 25 seeds were placed in 50 mm Petri dishes, on two sheets of Whatman no. 1 filter paper, to which 1.5 mL of test solution containing different NaCl concentrations was added in a LMS 250 incubator (LMS, Kent, UK) with a light flux density of 7 µmol m^−2^ s^−1^. Germination was recorded daily for 2 weeks until no further germination was recorded—for D11, D16 and D20 after 15 days, and for D17, D18 and D19 after 13 days. Germination was defined as radicle emergence of 1 mm. Mean time to germination (MTG) in days was calculated as ∑*Dn*/∑*n*, where *n* is the number of seeds that germinated in day *D*, and *D* is the number of days from the start of germination (Pritchard and Miller 1995). After 2 weeks, seeds were washed in distilled water and the experiment was continued at the same temperature regimes for 15 days in distilled water to measure the recovery from salt stress.

### Optimization of seedling establishment

*Salicornia dolichostachya* seeds (collection D16) were sown into soil (Einheitserde*,* Einheitserdewerk Hameln-Tündern, Germany) in trays (Styrofoam 25 cm length × 15 cm width) with four replicates of 25 seeds for each treatment. Experiments were carried out in a greenhouse under controlled conditions of 22 ± 2 °C; artificial light was supplied by sodium vapour lamps (SON-T 400W, Philips Deutschland GmbH) to obtain a photon flux density in the greenhouse of at least 350 µmol m^−2^ s^−1^ during an 18 h day. Germination trays were covered with a transparent plastic sheet to maintain a high humidity of >80 %. To study the effect of salinity on germination in soil, four replicates of 25 seeds of *S. dolichostachya* seeds were subjected to five salinity regimes (0, 100, 200, 400 and 700 mM NaCl) achieved by the application of 150 mL of tap water per box per week containing the respective concentrations of NaCl. Successful germination was quantified after 30 days by counting visible seedlings when 20 seedlings from each treatment were transferred to pots (diameter 8 cm) filled with sand. The treatments were continued by the application of 40 mL of tap water per pot per week containing the respective concentrations of NaCl.

The three salinity treatments with the highest germination percentages (0, 100 and 200 mM NaCl) were used to determine the optimal salinity for seedling establishment. An initial harvest was made after 68 days and the final harvesting after 96 days. Fresh and dry biomass of shoots and roots of five plants for each condition were measured separately. For dry biomass, plant parts were kept at 110 °C (Cabinet dryer 600, Memmert GmbH + Co, KG Schwabach, Germany) until a stable weight was obtained.

### Cultivation of *Salicornia* spp. to harvestable size

To determine the optimal growth conditions for the cultivation of *Salicornia* spp. as a vegetable crop, experiments were performed with different culture media (hydroponics with or without sand as a supporting medium), salinity levels and species. The experiments were conducted in a greenhouse under conditions described in the previous section (the 18 h day length prevented early flowering). Each experiment was started with seed material sown on sand and then treated with Hoagland solution (Epstein 1972) containing 50 mM NaCl for 2 months. Plants were then adapted to high salinity in the artificial sea water used in the experiments by adding NaCl up to the respective conductivity during the 2 weeks before starting the experiments. The containers used for growing the plants had dimensions of 600 × 400 × 425 mm, length by width by height (Erwin Sander Elektroapparatebau GmbH, Uetze-Eltze, Germany) (see also Buhmann and Papenbrock 2013*a*).

The cultivation of two different species, *S. dolichostachya* (collection D16 and D19) and *S. ramosissima* (collection D8), was compared in sand culture. Containers were filled with gravel up to 10 cm from the base and sand up to 23 cm, and the two layers separated by a thin layer of horticultural fleece. Culture solution, ∼26 L of artificial sea water (Seequasal GmbH, Münster, Germany), was added per container and supplemented with a modified Hoagland solution (Epstein 1972) containing MoNa_2_O_4_ instead of MoO_3_ and NaFe-EDTA instead of NaFe-DTPA [**see Supporting Information**]. Water circulation was established through a drainage pipe in the gravel layer, a pump (Aquabee UP 300, Aquabee Aquarientechnik, Zerbst, Germany) and a sprinkling irrigation system of two pipes with holes above the sand layer. The water in the containers was circulated throughout the system for 12 h during the day (a scheme is shown in Buhmann and Papenbrock 2013*a*). Additionally, the influence of salinity on the growth of *S. ramosissima* was investigated in sand culture, using the two (257 and 513 mM NaCl) concentrations of artificial sea water, equivalent to 15 and 30 PSU (Practical Salinity Units), respectively. Although the artificial sea water does contain NaCl, salinity measured in PSU is given as the NaCl concentration. To investigate the potential of repeated harvests from the same plant, shoots were cut 10 cm above the sand level after 5 and 15 weeks. In this experiment, nutrients were added again to the containers after 5 and 10 weeks. For hydroponic culture, the growth of *S. dolichostachya* was compared with the growth in sand culture. Containers were filled with 70 L of artificial seawater with a salinity of 15 PSU (equal to 257 mM NaCl) and modified Hoagland solution. A styrofoam sheet was used as a base to support the plants on the solution surface, and continuous aeration was established by using an air pump.

In all experiments, three containers were used as a replicate, with nine plants (2 months old) in each container. At the start of an experiment, the biomass (fresh and dry) of a sample of nine plants was measured. At the end of each experiment, all nine plants of each replicate were harvested and fresh and dry mass for shoots and roots were determined. For dry biomass, plant material was dried at 110 °C with repeated weighing until a stable weight was reached.

The pH in the containers was recorded weekly (Multi 350i pH/ISE-/Sauerstoffleitfähigkeits-Messgerät, WTW Technische Werkstätten GmbH, Germany).

### Statistical analysis

Statistical analysis was performed using GraphPad Prism version 6.00 for windows (GraphPad software, La Jolla, CA, USA, www.graphpad.com). The effects of two variables, salinity and temperature, on germination percentage and MTG (four replicates per treatment) were analysed using two-way analysis of variance. In the case of investigation of the effects of one variable, salinity, on germination and biomass production (five replicates per treatment), data were analysed using one-way ANOVA. For multiple comparisons, Tukey's test was performed and the level of *P* < 0.05 was considered as significant for all statistical analyses.

## Results

### Genetic characterization of *Salicornia* spp.

**Figure 1.**
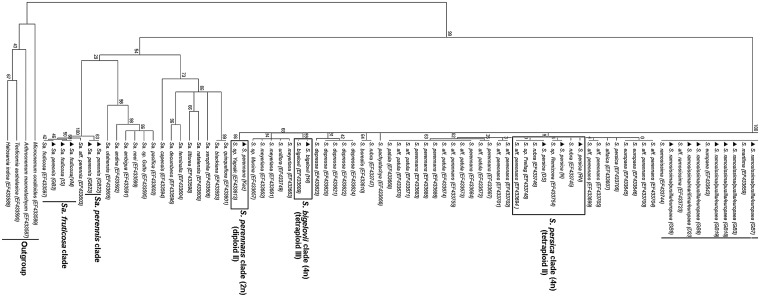

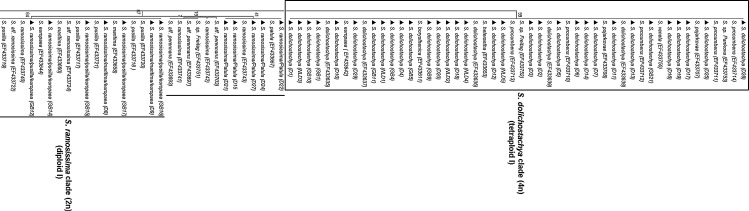
Maximum likelihood tree based on 164 ETS sequences; 62 ETS sequences are from collections used in this study and 102 ETS sequences were included from the NCBI database. Filled triangle before species represents original sequences of this study, and alpha-numeric code after species represents the location of sample collection (refer to Table 1 for exact location of each sample). Accession number of reference ETS sequences from GenBank is represented in brackets after each species. Ploidy levels of each sample used in this study were taken from Kadereit *et al.* (2007).

External transcribed spacer sequence data were obtained from 62 individuals (for location and accession numbers see Table 1 and **Supporting Information**) and analysed via a tree-based approach including reference sequences of various *Salicornia* and *Sarcocornia* spp. submitted to GenBank by Kadereit *et al.* (2007) (Fig. 1). A mML tree was generated using the GTR + G (GTR) model with 1000 bootstrap values. Analysis of ETS sequences distinguished species of *Salicornia* and *Sarcocornia* and clearly resolved *Salicornia* as a monophyletic group (ML bootstrap [mlbs] = 100 %). Out of 62 individuals, five were identified to belong to the genus *Sarcocornia*. Out of 57 individuals, 32 were grouped in the *S. dolichostachya* (tetraploid, 4*n* = 36) clade and 20 individuals in the *S. ramosissima* (diploid, 2*n* = 18) clade. Small clades of *Salicornia bigelovii* (tetraploid, 4*n* = 36), *Salicornia persica* (tetraploid, 4*n* = 36, contains three individuals) and *Salicornia perennans* (diploid, 2*n* = 18) were also present in the collections from Israel. The *Sarcocornia* lineage resolved into *Sarcocornia fruticosa* (octaploid, 8*n* = 72) and *Sarcocornia perennis* (diploid, 2*n* = 18). Ploidy levels of each sample used in this study were taken from Kadereit *et al.* (2007).

Analysis of ETS sequences clearly resolved *S. dolichostachya* at the molecular level but a clear distinction between *S. ramosissima/pusilla/patula* and *europaea* was not obtained. However, the ML analysis separated all individuals into different clades. The *Salicornia dolichostachya* clade (first tetraploid clade according to Kadereit *et al.* (2007)) contained the maximum number of individuals from south of Caeciliengroden (Jade Bay, Germany) and Wieringen (the Netherlands) (Table 1), which suggests that there is very little species diversity along the transect south of Caeciliengroden (Jade Bay) and from the Wieringen collection points. *Salicornia dolichostachya* was found at all five points along the transect at Caeciliengroden, while *S. ramosissma* was identified only at the points furthest away from the sea shore. At all collection sites in Germany and the Netherlands, *S. dolichostachya* was the most common species.

To verify the grouping of the ETS sequences produced in this study, sequences of other species from the *S. dolichostachya* clade published in GenBank (*S. borysthenica*, *S. europaea*, *S. pojarkovae*, *S. procumbens*, *S. heterantha*, *S. veneta* and *S. pankava*) were included in the phylogenetic analysis and their position in the trees supported the positioning of the newly produced sequences (Fig. 1).

The second clade in the ML tree was *S. ramosissima* (Diploid I); this clade contained individuals from Jade Bay, Germany and Great Britain (Fig. 1). External Transcribed Spacer sequences did not produce a clear resolution between different species of this clade; again additional ETS sequences were taken from GenBank, but the ML tree clearly showed it as monophyletic. However, the *S. ramosissima* clade was not as well supported (Mlbs = 67 %) as the *S. dolichostachya* clade (Mlbs = 99 %). A few samples, from the furthest point from seashore in our collection north of Caeciliengroden (Jade Bay, Germany), were in this clade. *Salicornia ramosissima* (diploid) clade comprised several other species viz*. S. patula, S. maritima, S. pusilla, S. europaea* and *S. aff. perennans*. The ETS sequences were not variable enough to resolve these species at the molecular level.

The second tetraploid clade in the ML tree was the *S. persica* clade. This clade included three accessions from Israel and clearly resolved them as *S. persica* as well as several other species (*S. aff. perennans*, *S. rubra*). Maximum likelihood analysis clearly showed this clade as monophyletic, but this was not as well supported as the *S. dolichostachya* clade. There was a third tetraploid clade, where *S. bigelovii* formed a well-supported (Mlbs = 99 %) monophyletic group. *Salicornia bigelovii* and its sister clade *S. depressa* received high statistical support of 99 and 91 %, respectively.

*Salicornia perennans* (Kaz) formed a separate clade with statistical support of 86 % and showed a very divergent lineage from other *Salicornia* clades. Reference ETS sequences of *S. perennans* from the GenBank database are grouped in the *S. persica* clade and other sister clades of *S. persica.* The *S. perennans* (Kaz) sample was collected from a highly saline area near the shore of the Aral Sea in Kazakhstan. In the ML tree, *S. perennans* and the reference sample *S.* sp. Yaprak, both from Kazakhstan, have been placed in a separate group away from other *S. perennans* reference samples.

*Sarcocornia* was resolved as a distinct lineage and was separated into two main *Sarcocornia* clades, *Sa. fruticosa* clade and *Sa. perennis. Sarcocornia fruticosa* and *Sa. perennis* were grouped on a separate branch (statistically highly supported, Mlbs = 100 %) in comparison to other *Sarcocornia* spp.

### Heteromorphism of seeds and seed viability

The seeds used for germination studies (D11 and D16 to D20) were all collected from single plants identified as *S. dolichostachya* (Table 1). *Salicornia dolichostachya* produces more than about 2000 seeds per plant, but this varied between collections, with D18 producing the highest number (3783 ± 1381) and D16 the lowest number (2133 ± 1268) of seeds per plant (Table 2). Based on the assessment by Ungar (1979) of seed dimorphism in *S. europaea*, seeds of *S. dolichostachya* were separated into two groups, small and large, based on their size (≤1.4 and ≥1.5 mm, respectively). There was a significant difference (*P* < 0.0001) between the mass of small and large seeds (Table 2), with small seeds ranging from 0.129 ± 0.031 to 0.235 ± 0.030 mg, and large seeds from 0.204 ± 0.044 to 0.332 ± 0.053 mg.

**Table 2.** Number of seeds per plant (five plants per collection were counted), and mean mass of one small and large seed. Values are means ± standard deviation.

Collection number	Number of seeds per plant	Mean mass of one small seed (mg)	Mean mass of one large seed (mg)
D11	3320 ± 1232	0.176 ± 0.025	0.316 ± 0.058
D16	2133 ± 1268	0.162 ± 0.043	0.229 ± 0.036
D17	2779 ± 1213	0.235 ± 0.030	0.332 ± 0.053
D18	3783 ± 1381	0.129 ± 0.031	0.204 ± 0.044
D19	2737 ± 840	0.173 ± 0.065	0.306 ± 0.050
D20	2333 ± 1909	0.130 ± 0.042	0.275 ± 0.028

Small and large seeds from all collections of *S. dolichostachya* showed high viability ranging from 90 to 100 % according to the tetrazolium test (data not shown). Lowest viability (90 %) was observed in smaller seeds of D20, while large seeds showed 100 % viability. In contrast, 95 % viability was observed in large seeds of D11, while small seeds showed 100 % viability. In the case of D16, small and large seeds both showed 95 % viability. For other collections (D17, D18 and D19) 100 % viability was observed in both small and large seeds. Therefore, seed size had no consistent effect on seed viability and there is no generalized preferable seed size for the culture of *Salicornia* spp.

### Effect of temperature and salinity on germination of different ecotypes

For all six seed collections (D11 and D16–D20), two-way ANOVA confirmed (*P* < 0.0001) a significant effect of temperature and salinity on germination (Table 3). The interaction of temperature and salinity was significant for all collections except D20 (Table 3).

**Table 3.** Results of two-way ANOVA for germination data. Collection number D11 was collected from the nearest point to the seashore and other collections (D16–D20) were collected along the transect from the nearest point (D16) to the furthest point (D20).

No.	Variable factor	Sum of square	Degree of freedom	Mean square	*P* value
D11	Interaction	4258	20	212.9	0.0026
	Temperature	4558	4	1139	<0.0001
	Salinity	11 460	5	2292	<0.0001
D16	Interaction	7077	20	353.9	0.0005
	Temperature	39 371	4	9843	<0.0001
	Salinity	32 841	5	6568	<0.0001
D17	Interaction	2463	5	492.5	0.0056
	Temperature	2241	1	2241	0.0001
	Salinity	9207	5	1841	<0.0001
D18	Interaction	1335	5	267.0	0.0461
	Temperature	1875	1	1875	0.0002
	Salinity	14 442	5	2888	<0.0001
D19	Interaction	4727	5	945.4	<0.0001
	Temperature	3675	1	3675	<0.0001
	Salinity	13 562	5	2712	<0.0001
D20	Interaction	3761	20	188.1	0.1122
	Temperature	3263	4	815.7	0.0001
	Salinity	29 323	5	5865	<0.0001

**Figure 2.**
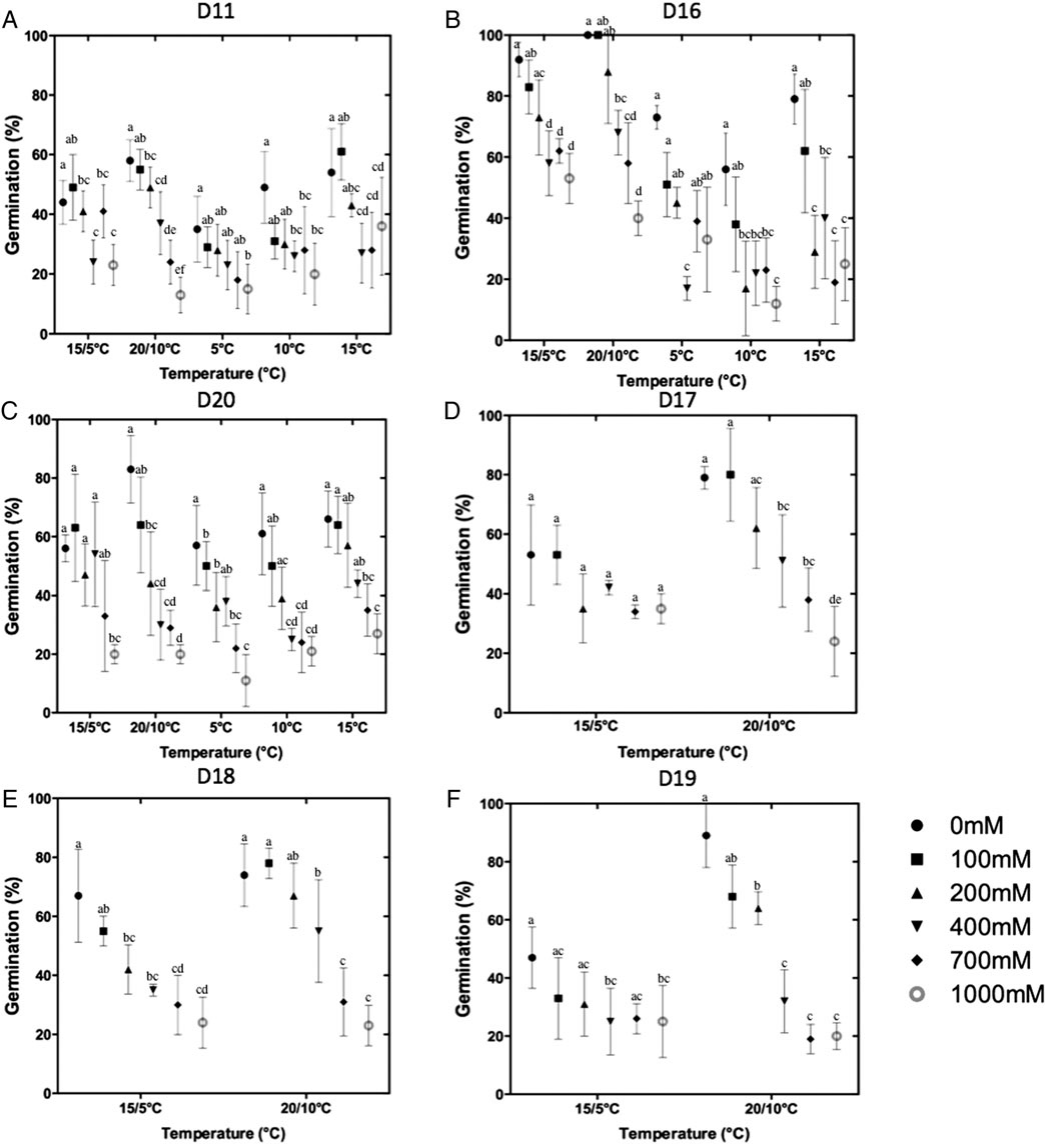
Graphs showing influence of the temperature and salinity on the germination behaviour of *Salicornia* spp. seeds, mixtures containing both small and large seeds, from six different collections (D11, D16, D17, D18, D19 and D20). Germination percentage was measured after 15 days for D11, D16 and D20, while for D17, D18 and D19 germination (%) was measured after 13 days. Tukey's test was used for multiple comparisons under different salinity and temperature conditions. Values with the same letters, under the same temperature are not significantly different at *P* > 0.05.

Of all the collections, D16 (collected near to the seashore) showed the highest mean germination percentage at all temperature and salinities (Fig. 2). The maximum germination percentage for D16 (100 %) occurred in 0 and 100 mM NaCl at 20/10 °C, while 700 and 1000 mM NaCl reduced germination to 53 and 40 % (at the same temperature) , respectively. For D17, D18, D19 and D20 (collected furthest from the seashore), maximum germination (79, 74, 89 and 83 %, respectively) was again recorded at 20/10 °C at 0 and 100 mM NaCl. Although the germination percentage was found to decrease with increasing salt concentration, all six collections showed ≥10 % germination at the highest level of salt stress (1000 mM NaCl). Generally, the temperature treatment 20/10 °C showed the best germination results under non- or low-saline conditions; for D11, 15 °C was equally suitable.

The MTG (in days) was calculated to determine the vigour for all six seed collections (Table 4). All collections tended to have the fastest MTG at 15/5 °C and 20/10 °C, and the effect of salinity on MTG at the same temperature was not significant for most of the collections (Table 4). Two-way ANOVA (*P* < 0.05) analysis of MTG data showed that any effect of salinity, temperature and their interaction was specific to each collection (Table 5). In summary, for all collections the germination percentage was highest at lower salinities and a slower MTG was shown for treatments where the temperature was constant during both the light and dark periods.

**Table 4.** The influence of temperature and salinity on MTG (in days) of *Salicornia* spp. seeds from six different collections. Values are means ± standard deviation. Tukey′s test was used for multiple comparisons under different salinity and temperature conditions. Values with the same letters within a temperature column are not significantly different at *P* > 0.05.

No.	Salinity (mM)	15/5 °C	20/10 °C	5 °C	10 °C	15 °C
D11	0	2.08 ± 0.70^a^	6.83 ± 3.87^a^	3.80 ± 1.15^a^	3.41 ± 0.68^a^	5.25 ± 1.71^a^
	100	3.98 ± 1.69^a^	4.38 ± 0.81^ab^	4.76 ± 1.12^a^	4.18 ± 2.20^a^	5.90 ± 2.17^a^
	200	3.27 ± 1.59^a^	4.36 ± 0.93^ab^	5.43 ± 4.39^a^	5.76 ± 2.11^a^	6.50 ± 2.12^a^
	400	1.79 ± 1.08^a^	6.31 ± 1.98^ab^	5.74 ± 1.01^a^	7.83 ± 2.51^a^	4.51 ± 2.96^a^
	700	1.21 ± 0.50^a^	3.28 ± 2.24^ab^	8.21 ± 3.14^a^	5.53 ± 3.73^a^	5.61 ± 4.17^a^
	1000	1.39 ± 0.19^a^	1.60 ± 0.49^bc^	6.61 ± 3.38^a^	5.57 ± 3.32^a^	4.40 ± 0.94^a^
D16	0	2.66 ± 0.21^a^	2.61 ± 0.65^a^	5.28 ± 0.99^a^	6.02 ± 0.98^a^	5.61 ± 0.73^a^
	100	3.10 ± 0.93^a^	2.73 ± 1.20^a^	2.96 ± 1.06^ab^	7.86 ± 1.58^a^	7.61 ± 1.69^ab^
	200	4.55 ± 0.28^a^	3.84 ± 1.32^a^	4.41 ± 1.63^a^	3.87 ± 3.92^ab^	5.51 ± 1.75^ab^
	400	2.12 ± 0.82^a^	3.37 ± 0.66^a^	8.15 ± 4.61^ac^	7.96 ± 1.43^a^	5.77 ± 3.04^a^
	700	1.37 ± 0.27^a^	2.22 ± 0.66^a^	3.36 ± 1.78^ab^	9.39 ± 3.74^cd^	5.42 ± 2.95^ac^
	1000	2.06 ± 0.53^a^	1.69 ± 0.25^a^	4.49 ± 2.59^ab^	12.7 ± 2.98^d^	10.0 ± 4.10^d^
D20	0	3.84 ± 0.59^a^	3.50 ± 0.73^a^	5.81 ± 0.85^a^	4.44 ± 1.49^a^	4.03 ± 1.20^a^
	100	4.99 ± 1.68^ab^	4.77 ± 1.12^a^	5.03 ± 1.67^a^	4.09 ± 0.97^a^	5.59 ± 1.05^ab^
	200	4.11 ± 0.86^a^	3.50 ± 1.31^a^	3.16 ± 1.59^a^	5.06 ± 1.98^a^	6.57 ± 2.06^ab^
	400	1.93 ± 1.34^a^	2.98 ± 0.41^a^	2.64 ± 1.03^a^	4.17 ± 3.31^a^	9.57 ± 1.41^c^
	700	1.87 ± 0.55^a^	2.21 ± 1.16^a^	4.29 ± 2.04^a^	5.40 ± 3.87^a^	6.18 ± 1.61^a^
	1000	1.39 ± 0.28^ac^	1.60 ± 0.43^a^	4.79 ± 3.25^a^	4.23 ± 1.31^a^	5.33 ± 1.67^ad^
D17	0	3.60 ± 0.98^a^	4.42 ± 0.76^a^			
	100	3.79 ± 1.25^a^	4.35 ± 0.49^a^			
	200	3.52 ± 2.66^a^	5.61 ± 0.69^ab^			
	400	3.31 ± 1.16^a^	3.32 ± 0.85^a^			
	700	2.80 ± 0.19^a^	2.50 ± 0.72^a^			
	1000	2.58 ± 0.09^a^	3.23 ± 1.65^ac^			
D18	0	3.33 ± 1.13^a^	3.43 ± 0.54^a^			
	100	3.26 ± 0.65^a^	4.01 ± 0.90^a^			
	200	2.39 ± 0.95^a^	3.69 ± 0.84^a^			
	400	2.64 ± 1.11^a^	4.32 ± 1.63^a^			
	700	2.32 ± 0.46^a^	3.68 ± 1.32^a^			
	1000	3.47 ± 2.15^a^	2.02 ± 0.17^a^			
D19	0	2.88 ± 1.09^a^	4.08 ± 0.91^a^			
	100	3.31 ± 1.58a	4.63 ± 1.20^ab^			
	200	3.32 ± 1.86^a^	5.24 ± 1.15^ac^			
	400	2.06 ± 1.13^a^	4.40 ± 0.90^ad^			
	700	2.45 ± 0.60^a^	1.61 ± 0.77^ae^			
	1000	1.73 ± 0.65^a^	1.63 ± 0.63^ae^

**Table 5.** Results of two-way ANOVA for MTG (in days) data.

No.	Variable factor	Sum of square	Degree of freedom	Mean square	*P* value
D11	Interaction	180.05	20	9.025	0.0044
	Temperature	24.14	5	4.828	0.4730
	Salinity	189.1	4	47.28	<0.0001
D16	Interaction	297.9	20	14.90	<0.0001
	Temperature	54.06	5	10.81	0.0335
	Salinity	532.8	4	133.2	<0.0001
D17	Interaction	6.282	5	1.366	0.4296
	Temperature	22.26	5	4.451	0.0155
	Salinity	4.928	1	4.928	0.0650
D18	Interaction	13.40	5	2.681	0.0815
	Temperature	4.57	5	0.915	0.6025
	Salinity	4.68	1	4.681	0.0605
D19	Interaction	15.22	10	1.522	0.5319
	Temperature	44.12	5	8.823	0.0007
	Salinity	18.25	2	9.103	0.0079
D20	Interaction	148.9	20	7.444	0.0007
	Temperature	22.93	5	4.587	0.1564
	Salinity	162.8	4	40.69	<0.0001

### Recovery of ungerminated seeds

For all six collections, the germination percentage following recovery in distilled water was >87 % and for most it was 100 % (Table 6). For D17, D18 and D19, 100 % recovery occurred at 20/10 °C in seeds from all salt concentrations except 700 mM for D17. Similarly, for D16 and D20 at 15 °C recovery was 100 %. A two-way ANOVA confirmed that there was no significant effect of temperature and salinity (salt concentration in which seeds were first placed) on the recovery percentage. However, recovery of seeds from salt treatment depended on the specific ecotypes as D17, D18 and D19 showed almost 100 % recovery at 15/5 °C while D11, D16 and D20 showed only 87–97 % of recovery at 15/5 °C. This result confirms that seeds which did not germinate under high or low salinity were viable and germination occurred when seeds were placed under non-saline conditions.

**Table 6.** Recovery percentage of ungerminated *Salicornia* seeds, after 14 days, when placed in non-saline solution from different salt concentrations. Values are means ± standard deviation. An asterisk (*) denotes that all seeds germinated at the given salt concentration, therefore no recovery data are presented.

No.	Salinity (mM)	15/5 °C	20/10 °C	5 °C	10 °C	15 °C
D11	0	98.1 ± 3.84	100 ± 0.00	98.3 ± 3.33	100 ± 0.00	100 ± 0.00
	100	92.3 ± 9.15	100 ± 0.00	97.3 ± 3.16	100 ± 0.00	93.7 ± 12.51
	200	93.3 ± 5.49	100 ± 0.00	98.6 ± 2.77	98.5 ± 2.95	96.4 ± 7.14
	400	94.9 ± 7.07	100 ± 0.00	96.2 ± 2.53	100 ± 0.00	100 ± 0.00
	700	94.2 ± 1.72	100 ± 0.00	98.8 ± 2.28	100 ± 0.00	97.36 ± 5.26
	1000	96.1 ± 4.68	98.80 ± 2.38	97.6 ± 2.80	100 ± 0.00	97.5 ± 5.12
D16	0	87.5 ± 4.89	*	89.6 ± 12.48	100 ± 0.00	100 ± 0.00
	100	91.4 ± 10.16	*	100 ± 0.00	100 ± 0.00	100 ± 0.00
	200	93.7 ± 12.51	100 ± 0.00	100 ± 0.00	100 ± 0.00	100 ± 0.00
	400	92.14 ± 10.88	92.04 ± 12.14	100 ± 0.00	97.6 ± 4.76	100 ± 0.00
	700	95.0 ± 5.77	87.35 ± 9.18	100 ± 0.00	100 ± 0.00	100 ± 0.00
	1000	98.2 ± 3.57	100 ± 0.00	98.2 ± 3.57	93.53 ± 19.31	100 ± 0.00
D20	0	97.5 ± 5.00	100 ± 0.00	100 ± 0.00	97.2 ± 5.55	100 ± 0.00
	100	100 ± 0.00	100 ± 0.00	100 ± 0.00	100 ± 0.00	100 ± 0.00
	200	96.7 ± 3.73	100 ± 0.00	100 ± 0.00	100 ± 0.00	100 ± 0.00
	400	94.32 ± 7.20	98.07 ± 3.84	100 ± 0.00	100 ± 0.00	100 ± 0.00
	700	88.30 ± 6.23	100 ± 0.00	100 ± 0.00	100 ± 0.00	100 ± 0.00
	1000	96.30 ± 2.46	100 ± 0.00	100 ± 0.00	100 ± 0.00	100 ± 0.00
D17	0	100 ± 0.00	100 ± 0.00			
	100	100 ± 0.00	100 ± 0.00			
	200	100 ± 0.00	100 ± 0.00			
	400	100 ± 0.00	100 ± 0.00			
	700	100 ± 0.00	97.5 ± 5.00			
	1000	100 ± 0.00	100 ± 0.00			
D18	0	100 ± 0.00	100 ± 0.00			
	100	94.44 ± 11.12	100 ± 0.00			
	200	100 ± 0.00	100 ± 0.00			
	400	93.75 ± 12.51	100 ± 0.00			
	700	100 ± 0.00	100 ± 0.00			
	1000	100 ± 0.00	100 ± 0.00			
D19	0	100 ± 0.00	100 ± 0.00			
	100	100 ± 0.00	100 ± 0.00			
	200	100 ± 0.00	100 ± 0.00			
	400	96.74 ± 3.81	100 ± 0.00			
	700	100 ± 0.00	100 ± 0.00			
	1000	100 ± 0.00	100 ± 0.00			

### Influence of day length and salinity on seedling establishment

For germination experiments under controlled conditions, the use of Petri dishes is convenient. But for commercial production of *Salicornia* spp. Petri dishes are impractical and costly, and the use of a support medium is preferred. In pre-experiments, soil was found to be a promising medium for germination of seeds of *Salicornia* spp., although germination behaviour could differ from that in Petri dishes. Therefore germination of collection D16 on soil was carried out at five salinity regimens between 0 and 700 mM NaCl. Results support our finding that higher germination rates occurred at low salinity and high salinity (400 and 700 mM NaCl) inhibited the germination (Table 7). However, germination percentage was much lower in soil than in Petri dishes (Fig. 2) with the highest germination percentage (37 and 31 %) occurring at 100 and 200 mM NaCl soil salinity, respectively. The lowest germination percentage (8 %) was shown at 700 mM NaCl. One-way ANOVA (*P* < 0.0001) confirmed that day length (Table 7) did not affect germination significantly.

**Table 7.** Effect of salinity and day length on the germination of *S. dolichostachya* in soil media. Values are means ± standard deviation. Tukey's test was used for multiple comparisons under different salinity levels and day lengths. Values with different letters are significantly different (*P* < 0.05).

	Germination percentage
Salinity (mM)	
0	26 ± 7.7^a^
100	37 ± 6.0^ab^
200	31 ± 8.2^abc^
400	20 ± 7.3^acd^
700	8 ± 5.7^d^
Day length	
14 h	32 ± 2.8^a^
16 h	43 ± 1.4^a^
18 h	34 ± 8.5^a^

**Figure 3.**
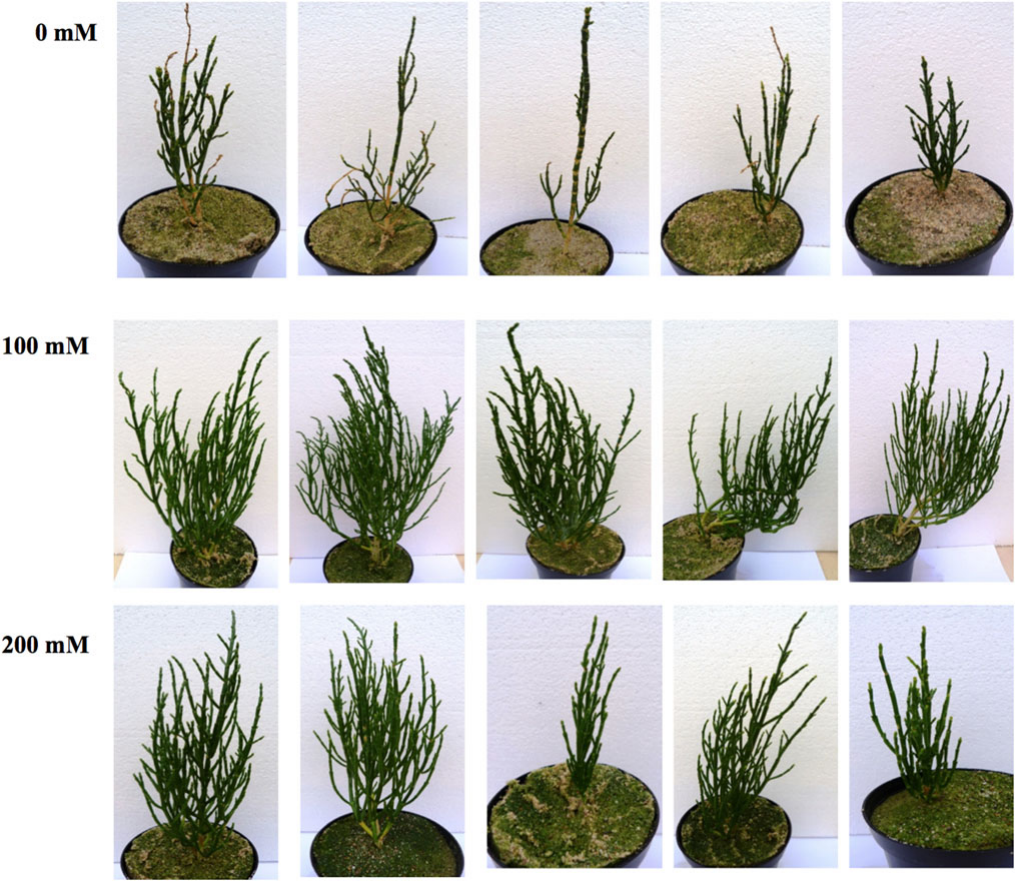
Photographs of 96-day-old *Salicornia dolichostachya* plants under 0, 100, and 200 mM salt (NaCl) stress.

An investigation of the effect of the three salinities with the highest germination percentages (0, 100 and 200 mM NaCl) on the establishment of *S. dolichostachya* showed the optimal salinity to be100 mM NaCl, growth (Table 8). One-way ANOVA showed that shoot and root biomass (both fresh and dry biomass) differed significantly between 100 and 200 mM, while there was no significant difference between 0 and 200 mM during the second harvest (Table 8). Plants grown at 100 mM produced more branches and were taller than plants under 0 and 200 mM NaCl (Fig. 3).

**Table 8.** Effect of salinity (0, 100 and 200 mM) on fresh and dry biomass of *S. dolichostachya* plants. Values are means ± standard deviation. First harvesting was carried out after 68 days and second harvesting after 96 days. Tukey's test was used for multiple comparisons. Values with different letters are significantly different (*P* < 0.05).

	Fresh biomass (g)			Dry biomass (g)		
	0 mM	100 mM	200 mM	0 mM	100 mM	200 mM
First harvest						
Shoot	2.81 ± 0.96^a^	5.28 ± 2.40^ab^	1.98 ± 1.15^ac^	N/A	N/A	N/A
Root	0.69 ± 0.38^a^	1.30 ± 0.65^ab^	0.30 ± 0.18^ac^	N/A	N/A	N/A
Second harvest						
Shoot	9.1 ± 6.9^a^	60.0 ± 21.9^b^	21.0 ± 13.2^a^	0.58 ± 0.27^a^	5.46 ± 1.95^b^	1.65 ± 1.23^a^
Root	2.5 ± 0.8^a^	14.8 ± 2.4^b^	5.8 ± 4.2^a^	0.44 ± 0.18^a^	1.80 ± 0.79^b^	0.71 ± 0.51^a^

To prevent early flowering, in spring/early summer time a day length of 14 h obtained by additional artificial light was sufficient as was observed during 90 days for *S. dolichostachya*. However, experiments in winter time and in climatic chambers revealed that longer periods of light, ∼18 h, were necessary to prevent flowering reliably (data not shown).

### Comparison of *S. dolichostachya* and *S. ramosissima*

The two species *S. dolichostachya* and *S. ramosissima* differ in their morphology. *Salicornia ramosissima* shows, in general, a more compact growth form than *S. dolichostachya* , with more branches of the stem and thus more biomass per unit volume, a trait that might be advantageous for commercial cultivation, as may be the fact that *S. ramosissima* is usually found under drier and less-saline conditions than *S. dolichostachya*. Consequently, having optimized the growth conditions for *S. dolichostachya*, we compared these with those optimal for *S. ramosissima.* Results showed a similar increase in fresh and dry biomass (in both shoot and root) for both species at an artificial sea water concentration equal to 257 mM NaCl (Table 9). Neither species showed any symptom of stress and both were healthy under the two salinities tested (257 and 513 mM). The increase in fresh and dry shoot and root biomass in *S. ramosissima* in the artificial sea water containing 257 mM NaCl was more than twice that containing 513 mM NaCl (Table 9).

**Table 9.** Increase in fresh and dry biomass in the experiments with sand and hydroponic culture (a), different salinities (b) and two harvests (c). D8, D16 and D19 represent the specimen voucher (refer to Table 1 for details). Collection D19 plants grown in the sand and hydroponic culture were harvested at the end of Week 6. D16 and D8 in sand culture were harvested at the end of Week 5. For the two-harvest experiment, first harvesting was carried out at the end of Week 5 and the second harvesting at the end of Week 15. Increase in biomass was calculated by subtracting the start biomass from the final biomass. Values with different letters (within the same column) are significantly different (*P* < 0.05). ^†^The artificial sea water does not only contain NaCl, but to make the salinity comparable with the other experiments, salinity originally measured in PSU is given as the NaCl concentration.

Experimental material	Substrate	Salinity (mM NaCl^†^)	Fresh biomass		Dry biomass	
			Shoot (g m^−2^)	Root (g m^−2^)	Shoot (g m^−2^)	Root (g m^−2^)
(a) Comparison between sand and hydroponic culture						
*S. dolichostachya* (D19)	Sand	257	858.92 ± 27.04^a^	47.49 ± 4.48^a^	68.38 ± 1.38^a^	9.81 ± 0.96^a^
*S. dolichostachya* (D19)	Hydroponic	257	1060.17 ± 118.55^b^	305.14 ± 48.40^b^	92.57 ± 9.40^b^	35.31 ± 5.89^b^
(b) Comparison between *S. dolichostachya* and *S. ramosissima* at different salinities						
*S. dolichostachya* (D16)	Sand	257	912.54 ± 232.97^a^	63.64 ± 6.48^a^	59.10 ± 17.27^a^	12.90 ± 3.53^a^
*S. ramosissima* (D8)	Sand	257	1031.82 ± 337.41^a^	60.79 ± 30.63^a^	65.11 ± 22.73^a^	13.38 ± 7.10^a^
*S. ramosissima* (D8)	Sand	513	433.39 ± 181.27^b^	11.15 ± 3.09^b^	27.58 ± 12.60^b^	1.96 ± 2.24^b^
(c) Biomass of *S. dolichostachya* after first and second harvest. An asterisk (*) denotes that root biomass data were not tested statistically because root biomass after first harvest was not measured						
*S. dolichostachya* (D16) (first harvest)	Sand	257	282.93 ± 174.86^a^		15.71 ± 11.01^a^	
*S. dolichostachya* (D16) (second harvest)	Sand	257	1578.60 ± 190.10^b^	122.38 ± 30.40*	213.21 ± 25.67^b^	23.61 ± 2.23*

### Cultivation of *Salicornia* spp. up to harvestable size

To determine the best culture medium for controlled and reproducible growth, *S. dolichostachya* (D16 and D19) and *S. ramosissima* (D8) seedlings were transferred to sand or hydroponic culture. For both species, the gain in shoot and root fresh and dry biomass was higher in hydroponics than in sand culture (Table 9). There was a continuous increase in pH with time in both culture systems: in hydroponics from 8.0 ± 0.11 at the beginning to 8.4 ± 0.07 at the end of Week 6 and in sand culture from pH 8.1 ± 0.04 to the final value of 8.3 ± 0.06. However, plants in hydroponics became slightly chlorotic while those in sand culture did not show any discoloration or signs of lack of nutrients in the medium.

We analysed the reaction to sequential harvesting of biomass 10 cm above the sand and the recovery and re-growth of the remaining plants in a two-harvest-experiment. After the first harvest at the end of Week 5, plants grew slowly and showed symptoms of stress such as yellowing of stems and drying of some branches. However, the plants recovered and gained biomass by the second harvest in Week 15 (Table 9).

## Discussion

### Identification of *Salicornia* spp. by molecular marker analysis demonstrates the importance of working with defined genetic material

For the use of *Salicornia* spp. as a crop plant, genetically defined material and well-characterized conditions for germination and growth are of great importance. Our results show that the application of one molecular marker, ETS, can define most *Salicornia* species and genotypes. When the plant material was originally collected, the respective specimens were identified by several plant scientists. However, the comparison of the initial identification based on morphology and the results obtained by ETS analyses were in agreement only in a few cases (data not shown). Since correct genotype determination is very important for selection and breeding we suggest the use of ETS analysis. Based on our data, a selection of genetically well-defined native *Salicornia* species for the commercial application can be started, probably supported in the future by more sensitive marker systems, such as amplified fragment-length polymorphism (AFLP) or microsatellite analysis.

The ML analysis based on ETS sequences obtained during this study clearly resolved *Salicornia* as a monophyletic group, in agreement with the molecular taxonomic studies by Kadereit *et al.* (2007). Both diploid (2*n* = 18) and tetraploid (2*n* = 36) species occur in the *Salicornia* genus (Shepherd and Yan 2003) with the *S. dolichostachya* clade forming a well-supported (Mlbs = 99 %) tetraploid monophyletic group. The *S. dolichostachya* clade is molecularly very uniform, and most species within this clade are difficult to separate morphologically and ecologically (Kadereit *et al.* 2007). However, surprisingly, in a recent study by Kadereit *et al.* (2012) *S. dolichostachya* and other species in the *S. dolichostachya* clade have been placed in the *S. procumbens* group, which contains both diploid and tetraploid species with five to nine variable nucleotides in the ETS sequence. It is assumed that two independent polyploidization events resulted in rapidly expanding tetraploid lineages. These lineages are able to grow in lower belts of the salt marshes than their diploid relatives (Kadereit *et al.* 2007).

The ML analysis we conducted showed that all three tetraploid clades (*S. dolichostachya*, *S. persica* and *S. bigelovii*) were not closely related; however, the *S. dolichostachya* clade was found to be closely related with the diploid *S. ramosissima* clade. Similarly, *S. persica* and *S. bigelovii* clades were found to be closely related with the diploid *S. ramosissima* and *S. perennans* clades, respectively. Similar results have been described by Kadereit *et al.* (2007): they reported that the *S. dolichostachya* and *S. persica* clades are most closely related to Southwest- and Central-Asian diploids. An increased sampling could reveal further relationships and distribution of polyploid lineages. As concluded by Kadereit *et al.* (2007), polyploid lineages are very successful in terms of range expansion, and most polyploid species are found in the lower and middle belts of salt marshes and often in monospecific stands under widely differing climatic conditions. This should be taken into account in the development of *Salicornia* species as crop plants.

The ETS sequences obtained during our study were not variable enough to resolve *S. pusilla*, *S. europaea* and *S. ramosissima* at the molecular level. However, these species have very different growth patterns and are morphologically distinct. Recent phylogenetic studies carried out by Kadereit *et al.* (2012) placed *S. pusilla* and *S. ramosissima* in the *S. europaea* group, and species with identical ETS sequences have been considered as a synonym of *S. europaea. Salicornia europaea* is a diploid (2*n* = 18) group and contains all *Salicornia* species having identical ETS sequences. Morphological characters for the *S. europaea* group may vary from one to three flowers per cyme, with lateral flowers one-third to two-thirds as large as the central flower (Kadereit *et al.* 2012). There are no differences among ETS sequences from *S. ramosissima*, *S. pusilla* and *S. europaea*, which clearly suggests that despite differences in growth form and number of flowers this molecular marker does not reveal a sufficient number of base differences for resolution (Kadereit *et al.* 2007). Analysis of ETS sequences also suggests that these species may have been named locally based on their morphological appearance, and require a better, uniform nomenclature system. It is therefore difficult to appreciate in the field that plants occurring in the same location and sharing a similar morphology possess different genotypes. At least in some cases, ETS is not variable enough to resolve morphologically distinct evolutionary units. Techniques such as AFLP fingerprinting could show the monophyly of more taxa.

In our study, *Salicornia* spp. were sampled from the wetter and often flooded area (nearest point from the seashore) to the drier area and less frequently flooded (furthest point from the seashore), and *S. dolichostachya* was the most common species at all collection sites in Germany and the Netherlands. This suggests that high physiological plasticity exists in *S. dolichostachya*, which has the potential to grow in low and high salinity as well as the different oxygen concentrations produced under flooded and drained conditions. The *S. dolichostachya* clade included individuals from Great Britain and confirms a wide-spread distribution of this species across three countries. *Salicornia ramosissima*, however, was mainly restricted to drier, less flooded areas and was absent from wetter, more frequently flooded areas (near to the seashore) in the collections.

External transcribed spacer sequences clearly resolved the two genera, *Salicornia* and *Sarcocornia*, at the molecular level and revealed *Salicornia* as a monophyletic taxon and *Sarcocornia* as paraphyletic (Kadereit *et al.* 2007). The perennial habit of *Sarcocornia perennis* also separates this genus from all species of *Salicornia* (Davy *et al.* 2001) and makes it very attractive for cultivation as a vegetable, as reported by Ventura *et al.* (2011*b*).

In conclusion, ML analysis based on ETS sequence data clearly supports the distinction between *Sarcocornia, Salicornia* and *Arthrocnemum* (Fig. 1, outgroup). Analysis of the ETS sequences also showed a clear distinction of *S. dolichostachya* at a molecular level, but in some cases ETS was not variable enough to resolve morphologically distinct clades (e.g. *S. pusilla*, *S. maritima* and *S. europaea* in the *S. ramosissima* clade). The results of this study also suggest that *S. dolichostachya* has a high physiological plasticity because it was found along a transect (from low to high elevations), while *S. ramosissima* was mainly confined to areas away from the sea shore.

### Seed dimorphism in *Salicornia* spp.

Results showed that *Salicornia* spp. produce dimorphic seeds with respect to seed size. Seeds of both small and large morphs were of similar viability, suggesting that the groupings were not related to seed maturity, where immature seeds may be smaller and less viable than mature seeds. Different seed size and deviation in the timing of germination among seeds of a single parent have been shown to affect the population biology of progeny by resulting in differential competitive ability, survivorship and reproductive output (Baker 1974; Baskin and Baskin 1976; Cideciyan and Malloch 1982). In some cases, for example, as demonstrated in *S. europaea*, seed polymorphism promotes the formation of a persistent seed bank, enabling the long-term survival of the species, particularly in a highly variable environment such as a salt marsh (Philipupilla and Ungar 1984). In our experiments, there were negligible differences in germination behaviour between the two morphs, meaning that the time-consuming and tedious work of separating seeds into small and large groupings is without any importance for the grower.

### Effect of salinity and temperature on germination

*Salicornia* spp. grow in highly saline areas, but their germination is inhibited by high salinity as shown by the results of this study. A similar effect of salinity on germination of *Salicornia* seeds has been described before (Ungar 1962; Hameed and Khan 2011). Generally, seed germination in European coastal halophytes occurs in early spring, when salinity is reduced by high soil moisture content, and temperatures are relatively low (Khan and Weber 1989). The results of this study showed that maximal germination was achieved at 20/10 °C, with comparable germination in some collections at a constant 15 °C, typical of spring temperatures for northern Europe.

As described by Levitt (1980) and Ungar (1978, 1995), germination of halophyte seeds is inhibited by high salinity, but salinity is not necessarily toxic as seeds will often recover and germinate when they are transferred to less-saline water (Levitt 1980; Ungar 1978, 1995). This was also observed in our experiments, with up to 40 % germination recorded even at 1000 mM NaCl at 20/10 and 15 °C (Fig. 2), and with high germination following recovery in distilled water. Seeds of *S. dolichostachya* are therefore highly tolerant to salinity during germination, but differences in the germination behaviour between the collections suggest that the optimization of the salinity level for germination requires evaluation by the grower.

### The effect of growth medium on seedling establishment

This experiment was performed to study the effect of salinity on seedling establishment of *Salicornia* spp. in soil. The results clearly showed that the germination percentage decreased with increase in salinity as in experiments in Petri dishes, although the germination percentage obtained in soil was much lower than that in Petri dishes at the same salinities. However, parameters other than the medium also differed between germination experiments. Germination in a Petri dish on moist filter paper showed the maximum potential of the seeds, but germination and seedling establishment in soil are important for commercial production as transplantation is too work intensive for commercial application.

As reported by Ventura *et al.* (2011*b*), critical day length (required to inhibit flower induction) differs between genotypes and ecotypes, with species originating from northern latitudes needing longer days (18 h) to prevent flowering, while for *Salicornia* genotypes, from further South in Israel, a continuous 13.5 h day length completely inhibited the onset of reproductive growth. York *et al.* (2000) also reported a similar observation that flower induction of *S. bigelovii* populations from northern latitudes (∼38°N) was more sensitive to short-day treatments than their Southern *Salicornia* spp. counterparts (∼28°N). Our own unreported data showed that a continuous illumination of *S. dolichostachya* seedlings for 14 h was sufficient to prevent plants from flowering. For a grower, the choice of genotype and consequent day length may be a significant issue.

### Optimization of cultivation conditions—effect of salinity on biomass production

*Salicornia dolichostachya* is a salt-tolerant species whose growth was optimal at 100 mM. Similar results, but with different optima for different genotypes have been reported by others: 200 mM NaCl for *S. persica* (Ahmad *et al.* (2013); 170–340 mM NaCl for *S. europaea*, *S. bigelovii*, *S. herbacea* and *S. brachystachya* (Cooper (1982); 200 mM NaCl for *S. brachiata* (Parida and Jha 2012). In contrast to our data, Katschnig *et al.* (2013) observed that 300 mM NaCl was optimal for *S. dolichostachya* in hydroponic cultures. Variation in optimal salt concentration for the same species could be due to variable conditions in a greenhouse such as substrate, temperature, day length and relative humidity. Nevertheless it is clear that *Salicornia* spp. requires salt for optimal growth, although the precise concentration to be used in a commercial operation would need to be determined. Leaf succulence and ion homoeostasis are discussed as the main reasons for salt-stimulated growth (Flowers *et al.* 1977; Lv *et al.* 2012; Katschnig *et al.* 2013) but the molecular basis for an optimal salt concentration is still not clear.

### Optimization of cultivation conditions—effect of culture medium on biomass production

In our study biomass (both fresh and dry biomass) production of *S. dolichostachya* in hydroponic culture was a little higher than in sand culture, but in hydroponics plants showed some chlorosis. Iron (Fe) is a key component of chlorophyll biosynthesis, and a shortage of accessible Fe in the nutrient solution could have produced this chlorosis (Spiller *et al.* 1982; Imsande 1998; Kobayashi *et al.* 2007). Iron deficiency frequently occurs in plants grown under alkaline conditions because availability of Fe to the plant is reduced (Römheld 1987). The high pH of the artificial sea water used as culture medium might have challenged Fe uptake by the *S. dolichostachya* plants. Supporting experiments have been carried out on another halophyte (sea aster, *Aster tripolium* syn. *Tripolium pannonicum*, Ventura *et al.* 2013). Ventura *et al.* (2013) demonstrated a successful elimination of chlorosis in sea aster in a saline agricultural system on sand dune soils by the application of Fe-EDDHA. Fe-EDDHA is more stable than Fe-EDTA at high pH and can prevent plants from iron deficiency in an alkaline soil or culture solutions with high pH (Lucena and Chaney 2006). The chlorosis in hydroponic culture was likely increased by the absence of a solid substrate, which prevented the plants from establishing an acidified micro-environment to facilitate the uptake of Fe and other micronutrients. The addition of Fe-EDDHA to hydroponic cultures of *Salicornia* reduced the Fe deficiency (data not shown) and Fe is now routinely added to the hydroponic cultures in our laboratory.

In our multiple harvest experiment, the dry weight accumulated by *S. dolichostachya* at the second harvest was more than 10 times that at the first, an increase in dry matter of 213 g DW m^−2^ in 10 weeks (at an artificial sea water concentration similar to 257 mM NaCl), which is equivalent to 1.1 kg DW m^−2^ yr^−1^. Several researchers have reported a slightly higher yield potential of *Salicornia* spp. in constructed wetland experiments; e.g. a yield of 1.54 kg DW m^−2^ year^−1^ was reported by O'Leary and Glenn (1994), 1.2–2.4 kg DW m^−2^ year^−1^ by Lu *et al.* (2001) and 1.7 kg DW m^−2^ year^−1^ by Ventura *et al.* (2011*a*). In our study only nine plants were grown in each container, and yield per unit area could be increased by a higher planting density. Ventura *et al.* (2011*a*) reported a successful multiple harvest experiment where a 3-week harvest regime for a genotype of *S. persica* resulted in six harvests of ∼1.5.5 kg FW m^−2^ (∼15 kg FW m^−2^ yr^−1^). In our experiment the biomass accumulation differed considerably between the two harvests, and the recovery time of the plants after cutting was long. Suitability of *Salicornia* spp. for the application of multiple-harvest regimes might depend on the species and also on climatic conditions, but the time between harvests and the amount of nutrients added during the intervals could be optimized.

## Conclusion

Maximum likelihood analysis based on ETS sequence data showed a clear distinction at the molecular level between the *Sarcocornia* and *Salicornia* clades collected for this study, and between the taxa *S. dolichostachya, S. persica, S. europaea, S. bigelovii* and *S. perennans*. However, the ETS sequences were not able to resolve all morphologically distinct species. Further studies with an even larger number of samples using additional molecular markers such as AFLP, and morphological and cytological investigation are required to make a distinct and uniform nomenclature for existing *Salicornia* spp.

Temperature and salinity were important factors that affected the germination of seeds. The optimal salinity level for growth and biomass accumulation of *S. dolichostachya* was 100 mM, with good potential for cultivation in both hydroponic and sand cultures. At a salinity equivalent to 257 mM NaCl in artificial sea water, *S. ramosissima* produced a higher biomass than *S. dolichostachya* , but high salinity (artificial sea water concentration similar to 513 mM NaCl) inhibited the growth of *S. ramosissima* and *S. dolichostachya*.

Further investigation could be initiated on the basis of this study to promote an improved genotype of *S. persica,* for example, for commercial use. An improved genotype of *Salicornia* spp. having high yield, high salt tolerance and beneficial nutritional value could be developed through a selected breeding programme.

## Sources of Funding

The research in the Hannover laboratory and travelling was funded by the Deutsche Bundesstiftung Umwelt (AZ 27708) and by COST (STSM FA0901-041011-011415). The Royal Botanic Gardens, Kew, receive grant-in-aid from DEFRA.

## Contributions by the Authors

D.S. and A.K.B. conducted the experimental work, D.S., A.K.B., C.E.S. and J.P. analysed the data, and D.S., A.K.B., T.J.F., C.E.S. and J.P. wrote the manuscript.

## Conflicts of Interest Statement

None declared.

## Supporting Information

The following Supporting Information is available in the online version of this article –

**Table S1.** Published ETS sequence data used for the inference of the phylogenetic tree. Abbreviations: *S*., *Salicornia*; *Sa*., *Sarcocornia*.

**Table S2.** Modified Hoagland solution (NO_3_^−^ and PO_4_^−^ concentration) used for optimization of growth conditions.

Additional Information
