# Supplementary material for: Salicornia as a crop plant in temperate regions: selection of genetically characterized ecotypes and optimization of their cultivation conditions
Source: AoB Plants. 2014 Nov 10;6:plu071. doi: 10.1093/aobpla/plu071 (PMC4268490; doi:10.1093/aobpla/plu071)
Supplement: Additional Information [file supp_6_plu071_index.html]

Salicornia as a crop plant in temperate regions: Selection of genetically characterized ecotypes and optimization of their cultivation conditions — Salicornia as a crop plant in temperate regions: selection of genetically characterized ecotypes and optimization of their cultivation conditions — Additional Information 

# *Salicornia* as a crop plant in temperate regions: selection of genetically characterized ecotypes and optimization of their cultivation conditions

## Additional Information

Additional Information

**Files in this Data Supplement:**

- Additional Information Table 1 - docx file
- Additional Information Table 2 - docx file
