# Supplementary material for: Salicornia as a crop plant in temperate regions: selection of genetically characterized ecotypes and optimization of their cultivation conditions
Source: AoB Plants. 2014 Nov 10;6:plu071. doi: 10.1093/aobpla/plu071 (PMC4268490; doi:10.1093/aobpla/plu071)
Supplement: Additional Information [file supp_plu071_plu071supp_table1.docx]

**Supporting Information**

**File S2**. Modified Hoagland solution (NO_3_^-^ and PO_4_^-^ concentration) used for optimization of growth conditions.

| Compound | Molecular weight | Stock solution concentration | | Volume added to one liter of water | Concentration of nutrients | |
| --- | --- | --- | --- | --- | --- | --- |
|  | **[g/mol]** | **[mM]** | **[g/L]** | **[mL]** | **[mg/L]** | **[µmol/L]** |
| Macronutrients |  |  |  |  |  |  |
| MgSO_4_ x 7 H_2_O | 246.47 | 1000.00 | 246.48 | 1.0 | 246.48 | 1000.00 |
| KCl | 74.56 | 1000.00 | 74.56 | 6.0 | 447.36 | 6000.00 |
| NaNO_3_ | 84.99 | 4031.94 | 342.67 | 2.0 | 685.35 | 8063.88 |
| H_2_NaPO_4_ x H_2_O | 137.99 | 526.47 | 72.65 | 1.0 | 72.65 | 526.47 |
| Micronutrients |  |  |  |  |  |  |
| KCl | 74.56 | 25.00 | 1.86 | 2.0 | 3.73 | 50.00 |
| H_3_BO_3_ | 61.83 | 12.50 | 0.77 |  | 1.55 | 25.00 |
| MnSO_4_ x H_2_O | 169.01 | 1.00 | 0.17 |  | 0.34 | 2.00 |
| ZnSO_4_ x 7 H_2_O | 287.54 | 1.00 | 0.29 |  | 0.58 | 2.00 |
| CuSO_4_ x 5 H_2_O | 249.68 | 0.25 | 0.06 |  | 0.12 | 0.50 |
| MoNa_2_O_4_ x 2 H_2_O | 241.95 | 0.25 | 0.06 |  | 0.12 | 0.50 |
| Iron |  |  |  |  |  |  |
| C_10_H_16_FeN_2_NaO_8_ | 367.05 | 64.00 | 23.49 | 0.3 | 7.05 | 19.20 |
