# Supplementary material for: Salicornia as a crop plant in temperate regions: selection of genetically characterized ecotypes and optimization of their cultivation conditions
Source: AoB Plants. 2014 Nov 10;6:plu071. doi: 10.1093/aobpla/plu071 (PMC4268490; doi:10.1093/aobpla/plu071)
Supplement: Additional Information [file supp_plu071_plu071supp_table2.docx]

**Supporting Information**

**File S1.** Published ETS sequence data used for the inference of the phylogenetic tree. Abbreviations: *S*., *Salicornia*; *Sa*., *Sarcocornia*.

| **Taxa** | **Geographic source** | **GenBank accession number** |
| --- | --- | --- |
| *Arthrocnemum macrostachyum* | Turkey: Seyhan Prov., SE of Adana | EF433587 |
| *Halosarcia indica* | Australia: Western Mortlock River | EF433588 |
| *Microcnemum coralloides* | Spain: Aragon, Laguna de Guallar | EF433589 |
| *Tecticornia australasica* | Australia: Queensland, N of Townsville | EF433590 |
| *Sarcocornia ambigua* | USA: Massachusetts, Chatham | EF433591 |
| *Sa. andina* | Bolivia: Oruro Prov., 10 km N of Challapata | EF433592 |
| *Sa. blackiana* | Australia: Western Mortlock River | EF433593 |
| *Sa. capensis* | South Africa: Western Cape Prov., Gansbaai, Uilkraalsmond | EF433594 |
| *Sarcocornia* spp. Gutte & Moeller 9461a | Peru: Dpto. Cuzco, Huacarpay | EF433595 |
| *Sa. decumbens* | South Africa: Western Cape Prov., Gansbaai, Uilkraalsmond | EF433596 |
| *Sa. fruticosa* | Spain: Cantabria, Noja | EF433597 |
| *Sa. littorea* | South Africa: Western Cape Prov., Gansbaai, Uilkraalsmond | EF433598 |
| *Sa. neei* | Argentina: Patagonia, Chubut Prov., Cabo dos Bahias, Camarones | EF433599 |
| *Sa. natalensis* | South Africa: Western Cape, Kalbaskraal | EF433600 |
| *Sa. quinqueflora* | Australia: New South Wales, Karuah | EF433601 |
| *Sa. pacifica* | USA: California, Napa Valley near Vallejo | EF433602 |
| *Sa. aff. perennis* chen-572 | France: Loire-Atlantique, St-Nazaire | EF433603 |
| *Sa. terminalis* | South Africa: Northern Cape Prov., Namaqualand, Kamieskroon | EF433604 |
| *Sa. utahensis* | USA: Utah, Utah County | EF433605 |
| *Sa. xerophila* | South Africa: Western Cape Prov., Knersvlakte, Vanrhynsdrop, Quaggaskop | EF433606 |
| *Salcornia perennans* subsp. *altaica* | Russia: C Sib. Bot. Gard., seeds from Altai Mts. near Kosh Agach (M. Lomonosova 239/2002) | EF433607 |
| *S. borealis* | Canada: Yukon, between Whitehorse and Haines Junction | EF433610 |
| *S. borysthenica* | Ukraine: E Crimea, Arabatskaya Strelka | EF433611 |
| *Salicornia* spp.Yaprak 2004-1 | Turkey | EF433613 |
| *S. depressa* | USA: Rhode Island, Wakefield | EF433620 |
| *S. depressa* | USA: California, Napa Valley, San Francisco Bay | EF433621 |
| *S. depressa* | USA: Virginia, Gloucester Co., E of Williamsburg | EF433622 |
| *S. depressa* | USA: Virginia, Williamsburg E of Williamsburg | EF433623 |
| *S. depressa* | USA: New Jersey, Highlands, Sandy Hook | EF433624 |
| *S. dolichostachya* | France: Loire-Atlantique, La Turballe | EF433635 |
| *S. dolichostachya* | Germany: North Sea, Schleswig-Holstein, Buesum | EF433636 |
| *S. dolichostachya* | United Kingdom: Great Britain, Kent, Isle of Sheppey | EF433637 |
| *S. dolichostachya* | United Kingdom: Great Britain, Kings Lynn & West Norfolk, Brancaster Staithe | EF433638 |
| *S. dolichostachya* | Spain: Delta del Ebro, Punta del Falgar | EF433639 |
| *S. europaea* | Germany: Mecklenburg-Vorpommern, Zingst | EF433642 |
| *S. europaea* | Germany: North Sea, Schleswig-Holstein, Buesum | EF433643 |
| *S. europaea* | United Kingdom: Great Britain, East Sussex, Seaford | EF433644 |
| *S. europaea* | United Arab Emirates: Ras-al-Khaimah | EF433645 |
| *S. europaea* | Japan: Hokkaido, Abashiri, Lake Notoro | EF433646 |
| *S. procumbens* subsp. *heterantha* | Russia: Rostov Prov., Proletarsk, Manych Rivervall | EF433653 |
| *Salicornia* spp. Mucina 6928/3 | South Africa: Eastern Cape Prov., Gamtoos Rivermouth | EF433657 |
| *S. maritima* | Canada: Quebec, St. Andr | EF433658 |
| *S. maritima* | Canada: Prince Edward Island, SW of Summerside | EF433659 |
| *S. maritima* | Canada: Quebec, Magdalen Islands, Ile du Havre Aubert | EF433660 |
| *S. meyeriana* | South Africa: Western Cape, Cape Town, Milnerton, Rietvlei Nature Reserve (loc. class) | EF433661 |
| *S. meyeriana* | South Africa: Western Cape Prov., Gansbaai, Uilkraalsmond | EF433662 |
| *S. meyeriana* | South Africa: Western Cape Prov., Struisbaai, DeMond Nat. Res | EF433663 |
| *S. pachystachya* | South Africa: KwaZulu-Natal Prov., St. Lucia Wetland Park | EF433666 |
| *S. patula* | France: Camargue, Le-Grau-du-Roi | EF433667 |
| *S. patula* | Turkey: Canakkale, Gelibolu, Kavak Coragi | EF433668 |
| *S. aff. patula* Freitag 31.308 | Italy: SE Sicily, near Pachino | EF433670 |
| *S. aff. patula* Kadereit s.n. | Italy: Toscana, Marina di Grosseto | EF433671 |
| *S. aff. patula* Yaprak 2004-12 | Turkey: Sivas, Zara, Toeduerge Goelue | EF433672 |
| *S. aff. patula* Freitag 30.018 | Italy: Adriatic coast, Puglia, Gargano Peninsula, Vieste | EF433673 |
| *S. aff. patula* Freitag 31.313 | Italy: S Sicily, near Gela | EF433674 |
| *S. perennans* | Romania: Mures, Reghin, Ideci Bai | EF433683 |
| *S. perennans* | Romania: Mures, Sovata, Praid | EF433684 |
| *S. perennans* | Romania: Northern Transsylvania | EF433685 |
| *S. perennans* | Ukraine: W Crimea, Sakske lake near Saki | EF433686 |
| *S. perennans* | Kazakhstan: western, Kambash Lake near Small Aral Lake | EF433687 |
| *S. aff. perennans* Kadereit 2003/1 | Hungary: Hortobagyi Nemzeti Park | EF433693 |
| *S. aff. perennans* Lomonosova 277 | Russia: S Siberia, Irkutsk Prov., near Usole Sibirskoye | EF433694 |
| *S. aff. perennans* Wucherer 3a | Kazakhstan: western, Kambash Lake near Small Aral Lake | EF433697 |
| *S. aff. perennans* Yaprak 2004-19 | Turkey: Igdir, Tuzluca | EF433699 |
| *S. aff. perennans* Kuerschner & Sonnentag 00-626 | China: Nei Menggu Prov., 5 km S of Ejin Qi | EF433700 |
| *S. aff. perennans* Neuffer & Hurka 13624 | Russia: Altai Republic, NE of Aleysk at river Chaplaya | EF433701 |
| *S. aff.* perennans Zacharova 09.2003 | Russia: E Siberia, Yakutsk | EF433702 |
| *S. aff. perennans* Freitag 33.073 | Russia: Tuva, Uyuk vall. near, Arzhaan | EF433703 |
| *S. aff. perennans* Freitag 33.101 | Russia: Novosibirsk Prov., Karasuk distr., 20 km N of K | EF433704 |
| *S. aff. perennans* Freitag 33.061 | Russia: Tuva, Duz-Khol NW of Erzin | EF433705 |
| *S. persica* | Iran: Fars, N Tashk | EF433706 |
| *S. procumbens* subsp. *pojarkovae* | Russia: Karelia, White Sea, Kandalaksha Bay, Monastyrski Island | EF433707 |
| *S. procumbens* subsp. *pojarkovae* | Russia: Karelia, Loukhi distr., Peninsula Kindo | EF433708 |
| *S. procumbens* | Germany: North Sea, Schleswig-Holstein, Buesum | EF433710 |
| *S. procumbens* | Germany: North Sea, Schleswig-Holstein, Marne | EF433711 |
| *S. procumbens* | Germany: North Sea, Schleswig-Holstein, Meldorf | EF433712 |
| *S. procumbens* | Germany: North Sea, Schleswig-Holstein, Klanxbuel | EF433713 |
| *S. procumbens* | Germany: North Sea, Schleswig-Holstein, Buesum | EF433714 |
| *S. pusilla* | United Kingdom: Great Britain, Hampshire, Hayling Island | EF433717 |
| *S. pusilla* | United Kingdom: Great Britain, North Norfolk | EF433718 |
| *S. pusilla* | France: Manche, Portbail | EF433719 |
| *S. pusilla* | France: Somme, Le Crotoy | EF433720 |
| *S. pusilla* | France: Ille-et-Vilaine, Hirel | EF433721 |
| *S. aff. ramosissima* Teege 04F/0036 | France: Somme, Le Crotoy | EF433722 |
| *S. aff. ramosissima* Teege 04F/0106 | France: Somme, St-Valerie-sur-Somme | EF433723 |
| *S. aff. ramosissima* Teege 04F/0063 | France: Manche, Sainte-Marie-du-Mont | EF433724 |
| *S. ramosissima* | Spain: Delta del Anson, Marisma de Santona | EF433740 |
| *S. ramosissima* | Portugal: Algarve, Tavira, Olhao, Park of RiaFormosa | EF433741 |
| *S. ramosissima* | Spain: Cantabria, Noja, Playa de Ris | EF433742 |
| *S. ramosissima* | Spain: Alcaniz, Salada grande | EF433743 |
| *S. ramosissima* | France: Ille-et-Vilaine, Le-Vivier-sur-Mer | EF433744 |
| *S. rubra* | USA: Nevada, Nye County, Little Fish Lake (alt. 1970m) | EF433745 |
| *S. rubra* | USA: California, Redwood City | EF433746 |
| *S. rubra* | Canada: Quebec, Laprairie (introduced) | EF433747 |
| *Salicornia* spp. Freitag 33.074 | Russia: Tuva, 70 km NW Kyzyl, Beloye oz. near Arzhaan | EF433748 |
| *S. uniflora* | Namibia: Luederitz, Second Lagoon | EF433749 |
| *S. veneta* | Italy: Prov. Venetia, Lido di Jesolo | EF433750 |
| *Salicornia* spp. Freitag 19.812 | Greece: Peleponnissos, Patras, Varda Lagoon | EF433751 |
| *Salicornia* spp. Freitag 31.302 | Italy: SE Sicily, near Pachino | EF433752 |
| *Salicornia* spp. Pankova 5 | Russia: Astrakhan Prov, Narimanov | EF433753 |
| *Salicornia* spp. Remizowa & Sokoloff | Australia: Western Australia, Rottnest Island | EF433754 |
| *S. aff. perennans* Neuffer 10195 | Austria: Neusiedler See, near Ilmitz | EF433755 |
